# Evolution of Plant Architecture in *Oryza* Driven by the *PROG1* Locus

**DOI:** 10.3389/fpls.2020.00876

**Published:** 2020-06-23

**Authors:** Liyu Huang, Hui Liu, Junjie Wu, Ruoping Zhao, Yanxia Li, Getachew Melaku, Shilai Zhang, Guangfu Huang, Yachong Bao, Min Ning, Benjia Chen, Yurui Gong, Qingyi Hu, Jing Zhang, Yesheng Zhang

**Affiliations:** ^1^State Key Laboratory for Conservation and Utilization of Bio-Resources in Yunnan, Research Center for Perennial Rice Engineering and Technology of Yunnan, School of Agriculture, Yunnan University, Kunming, China; ^2^State Key Laboratory of Genetic Resources and Evolution, Kunming Institute of Zoology, Chinese Academy of Sciences, Kunming, China; ^3^Germplasm Bank of Wild Species, Kunming Institute of Botany, Chinese Academy of Sciences, Kunming, China; ^4^College of Agriculture and Biology Science, Dali University, Dali, China; ^5^BGI-Baoshan, Baoshan, China; ^6^Agricultural Biotechnology Directorate of the Ethiopian Biotechnology Institute, Addis Ababa, Ethiopia

**Keywords:** rice, *Oryza* species, *PROG1*, plant architecture, evolution, domestication

## Abstract

The genetic control of plant architecture in crops is critical for agriculture and understanding morphological evolution. This study showed that an open reading frame (ORF) of the rice domestication gene *PROG1* appeared 3.4–3.9 million years ago (Mya). Subsequently, it acquired a novel protein-coding gene function in the genome of *O. rufipogon* (~0.3–0.4 Mya). This extremely young gene and its paralogous C2H2 genes located nearby define the prostrate architecture of *O. rufipogon* and, thus, are of adaptive significance for wild rice in swamp and water areas. However, selection for dense planting and high yield during rice domestication silenced the *PROG1* gene and caused the loss of the *RPAD* locus containing functional C2H2 paralogs; hence, domesticated lines exhibit an erect plant architecture. Analysis of the stepwise origination process of *PROG1* and its evolutionary genetics revealed that this zinc-finger coding gene may have rapidly evolved under positive selection and promoted the transition from non- or semi-prostrate growth to prostrate growth. A transgenic assay showed that *PROG1* from *O. rufipogon* exerts a stronger function compared with *PROG1* sequences from other *Oryza* species. However, the analysis of the expression levels of *PROG1* in different *Oryza* species suggests that the transcriptional regulation of *PROG1* has played an important role in its evolution. This study provides the first strong case showing how a fundamental morphological trait evolved in *Oryza* species driven by a gene locus.

## Introduction

Unlike ancient genes, which often perform critical functions in species, newly evolved genes have been considered to be dispensable or to have minor biological functions (Miklos and Rubin, 1996; Zhang et al., 1999; Krylov et al., 2003). Previously, *de novo* origin of a protein-coding gene from non-coding sequences was even generally considered impossible (Jacob, 1977). Although recent works have reported that the existence of physiologically essential *de novo* genes and novel genes from gene duplication, to date, there are no reports of such genes controlling fundamental morphological traits (Chen et al., 2010; Li C.Y. et al., 2010; Li D. et al., 2010).

With the increasing amounts of genome data for *Oryza* species being reported, there species have become good model species for plant comparative genomics and phenotype studies, and the relationships between genotype and phenotype can be studied systematically in these taxa. Although a recent study identified several *de novo* genes based on expression at the RNA or protein level in *Oryza sativa* (Zhang L. et al., 2019), domestication genes that have been fixed in cultivated rice via a loss of function and their evolution progress have not been detected.

Asian cultivated rice (*O. sativa*) was domesticated ∼8,000–10,000 years ago (Sharma et al., 2000; Vaughan et al., 2008; Fuller et al., 2010). In the course of domestication, some traits, such as shattering (Konishi et al., 2006; Li et al., 2006), panicle architecture (Ishii et al., 2013; Zhu et al., 2013) and pericarp and hull colors (Sweeney et al., 2006; Zhu et al., 2011), were changed. In particular, plant architecture underwent extensive changes associated with efficient agricultural use, including the change from prostrate growth in the cultivated rice progenitor to an erect structure in both Asian and African cultivars. In previous studies, the monogenetic domestication gene *PROG1* in *O. sativa* and its paralog in *Oryza rufipogon* were cloned and identified as transcription factors based on their ∼90 bp C2H2-type zinc-finger motifs (Jin et al., 2008; Tan et al., 2008). These paralogs were found to have undergone strong artificial selection during the history of rice domestication (Jin et al., 2008; Tan et al., 2008; Wu et al., 2018). Although other genes controlling tiller angle and branching that play important roles in rice architecture, such as *Tiller Angle Controlling (TAC1)*, *LA1* (*LAZY1*), *IDEAL PLANT ARCHITECTURE1* (*IPA1*), and *OsTb2*, have been cloned in *O*. *sativa*, these genes have undergone selection only via artificial selection for high-density planting during domestication (Li et al., 2007; Yoshihara and Iino, 2007; Yu et al., 2007; Jiang et al., 2012; Lu et al., 2013; Lyu et al., 2020), with no evidence of a history of both natural and artificial selection.

In this study, the domestication gene *PROG1* was analyzed and identified as a young gene in *Oryza* that has driven the evolution of plant architecture. The open reading frame (ORF) of *PROG1* arose in *O. punctata* and evolved via natural selection into a prostrate-growth gene in *O. rufipogon*. More interestingly, *PROG1* was then functionally lost in *O. sativa* through artificial selection, which accompanied locus deletions (*RICE PLANT ARCHITECTURE DOMESTICATION*, *RPAD*) linked to the *PROG1* gene during artificial selection on architecture in the domestication of cultivated rice (Wu et al., 2018). Therefore, we hypothesize that the successive gain and loss of function of *PROG1* locus under natural and artificial selection, respectively, could result in variation of plant architecture during *Oryza* evolution.

## Materials and Methods

### *PROG1* Locus Sequence Alignment and Origin Analysis

Ten released genomes of *Oryza* species [*O. sativa* (Sasaki and International Rice Genome Sequencing Project, 2005), *O. glaberrima* (Wang et al., 2014), *O. longistaminata* (Zhang et al., 2015), *O. meridionalis* (Zhang et al., 2014), *O. glumaepatula* (Zhang et al., 2014)], *O. brachyantha* (Chen et al., 2013), *O. rufipogon* (Stein et al., 2018), *O. nivara* (Stein et al., 2018), *O. barthii* (Stein et al., 2018), *O. punctata* (Stein et al., 2018), and the *B. distachyon* genome (Vogel et al., 2010) were used in this study. The *PROG1* locus and its neighboring genes (two upstream and two downstream) were extracted from the *O. sativa* genome, and BLAST (Altschul et al., 1997) was used to obtain the genome sequences of remaining species, which were then annotated and aligned by using MEGA6 (Tamura et al., 2013).

### Phylogenetic Tree Construction and Divergence Time Estimation for 10 *Oryza* Species

Blastall (v2.2.21) (Altschul et al., 1997) with a threshold of “-e 1e-5” was used to align peptide sequences from the 10 *Oryza* species, and gene families were clustered by OrthoMCL (v1.4) (Li et al., 2003). From the identified single-copy gene families, 4-fold degenerated (4D) sites in the coding sequences of the genes were extracted and concatenated. Multiple sequence alignments were performed by MUSCLE (v3.7) (Edgar, 2004), and a phylogenetic tree with settings nst = 6, rates = invgamma and ngen = 1,000,000 was reconstructed using MrBayes (v3.1.2) (Ronquist and Huelsenbeck, 2003). To estimate divergence times among the 10 species, the program MCMCTree in PAML9 (v4.4) (Yang, 1997) with the parameters “clock = 3 and RootAge ≤ 0.1” was used. The divergence times were constrained by the fossil calibration times from TimeTree (0.4 Mya between *O. sativa* and *O. rufipogon*, 0.6–2.0 Mya between *O. punctata* and *O. meridionalis* and 9–15 Mya between *O. punctata* and *O. brachyantha*) (Hedges et al., 2006).

### Vector Construction and Rice Transformation

The *PROG1* promoter (1.5 kb) from *O. rufipogon* and coding sequences (CDSs) from *O. sativa*, *O. rufipogon* (Yuanjiang), *O. nivara*, *O. longistaminata*, *O. meridionalis*, *O. glumaepatula*, and *O. punctata* were amplified and inserted into the expression cassette pCAMBIA1300, and the *ProPROG1:PROG1-NOS* vectors were constructed. The recombinant plasmids were transferred into calli of the japonica rice cultivar Zhonghua11 (ZH11) by an *Agrobacterium tumefaciens*-mediated transformation method. The forward primer for the *PROG1* promoter was 5′-AATCAGCTCGAGCTAGGTCTTTG-3′, and the reverse primer for the *PROG1* promoter was 5′-GAAAGGAAAATGGGACAAGCTAT-3′. The forward primer for the *PROG1* CDS was 5′-ATGGATCCCTCATCGGCTTC-3′, and the reverse primer for the *PROG1* CDS was 5′-CTAGAGGCCGAGCTCGAGGA-3′.

## *Prog1* Locus Expression Analysis

Eight transcriptomes of *O. sativa* (Zhang et al., 2010), two transcriptomes of *O. nivara* and *O. barthii* (Wang et al., 2014), two transcriptomes of *O. punctata* (SRR1171006 and SRR1171007 in NCBI), two transcriptomes of *O. brachyantha* (Chen et al., 2013) and 11 transcriptomes of *B. distachyon* (Davidson et al., 2012) were downloaded from NCBI. Eight transcriptomes of *O. longistaminata* were obtained previously work (Zhang et al., 2015). RNA-seq reads from each sample were mapped to the corresponding reference genome with TopHat 2.0.3 with default parameters, and Cufflinks was then used to evaluate the FPKM values (Trapnell et al., 2012) of the *PROG1* locus and the internal control gene *Actin1*. To investigate the expression of the *PROG1* locus at the tiller base in *O. rufipogon*, *O. nivara*, *O. barthii*, *O. longistaminata* and *O. glumaepatula*, total RNA was extracted using TRIzol reagent (Invitrogen, United States) and reverse transcribed using the Revert Aid H Minus First Strand cDNA Synthesis Kit (Thermo Fisher Scientific) following the manufacturer’s instructions. qRT-PCR of *PROG1* was performed following the manufacturer’s instructions, and the *Tubulin* gene was used as the internal control. The forward primer for *PROG1* was 5′-GATCCCTCATCGGCTTCTT-3′, and the reverse primer for *PROG1* was 5′-GGAACAGCCTCACTTGCTTG-3′. The forward primer for *Tubulin* was 5′-GCTCCGTGGCGGTATCAT-3′, and the reverse primer for *Tubulin* was 5′-CGGCAGTTGACAGCCCTAG -3′.

### Field Experiment and Plant Architecture Survey

To investigate the plant architectures of *Oryza* species, 10 species (*O. sativa*, *O. rufipogon*, *O. nivara*, *O. glaberrima*, *O. barthii*, *O. longistaminata*, *O. meridionalis*, *O. glumaepatula*, *O. punctata*, and *O. brachyantha*) were grown in Xishuangbanna, Southwest China. The plant architectures were surveyed after 3 months.

### Tests of Selection on *PROG1* in *O. rufipogon* Populations

*PROG1* population data (Tan et al., 2008) for *O. rufipogon* were downloaded from NCBI and aligned using MEGA6 (Tamura et al., 2013). Tajima’s *D* test and Fu and Li’s test were conducted using DnaSP 5 (Librado and Rozas, 2009).

### Monte Carlo Simulations

Monte Carlo simulations were performed to determine the possibility that a random sequence could produce a C2H2 gene. Random sequences of 90 bp length (seed sequences) were generated accordance with the A, T, G, and C frequencies of the rice genome. The total number of seed sequences per simulation was a quotient of genome size and seed sequence length (T). A set of 100,000 simulations run with T seed sequences in each simulation was used. The number of C2H2 motifs for each seed sequence was counted by searching the motif pattern “Ø-X-C-X2,4,5-C-X3-Ø-X5-Ø -X2-H-X3,4-H” in all six reading frames (Klug and Schwabe, 1995). The distribution of the observed number of C2H2 genes was illustrated using kernel density estimation as implemented in R. The *p-*value (observed ≥ expected) was calculated by counting the frequencies of observed C2H2 genes that were equal to or larger than the expected C2H2 genes.

### Clustering of C2H2 Transcription Factor Genes in the Genome of *O. sativa*

The amino acid sequences of 189 C2H2 transcription factor genes (Agarwal et al., 2007) were divided into three types: full-length sequences, sequences with only the C2H2 motif, and sequences without the C2H2 motif. Multiple sequence alignments were performed using MUSCLE (v3.7) (Edgar, 2004). A phylogenetic tree was constructed with FastTree (2.1.10) (Price et al., 2009) and viewed with ETE (Huerta-Cepas et al., 2016).

## Results

### The ORF of *PROG1* Appeared in *O. punctata* and Has Experienced Different Evolutionary Fates in Different *Oryza* Species

**FIGURE 1:**
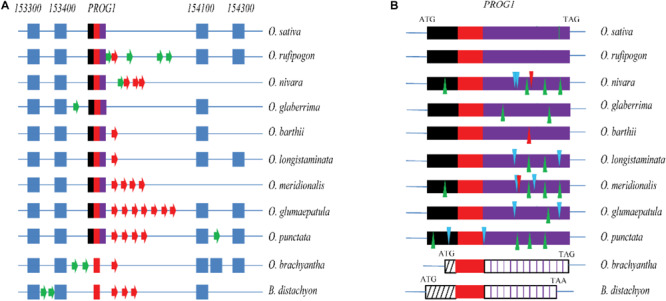
Alignment of the *PROG1* locus in different *Oryza* species and the outgroup *B*. *distachyon*. **(A)** Collinearity of the *PROG1* region in different species. *153300*, *153400*, *154100*, and *154300* represent the flanking genes *Os07g0153300*, *Os07g0153400*, *Os07g0154100*, and *Os07g0154300*, respectively. Blue blocks and tricolor (black, red, and purple) blocks represent neighboring genes and *PROG1* (the red blocks represent C2H2-type zinc-finger sequences). Arrows indicate other annotated genes in different genomes, green arrows indicate ORFs without homology with *PROG1*, and red arrows represent ORFs with C2H2 type zinc-finger sequences. **(B)** Alignment of the sequence of the *PROG1* locus in different species. Black, red, and purple blocks represent alignable 5′-termini, C2H2 type zinc-finger and 3′-terminal sequences, respectively; black and purple striped blocks represent unalignable 5′-termini and 3′-terminal sequences, respectively; green and blue triangles represent in-frame deletions and insertions, respectively. The red triangles (*O. barthii* and *O. meridionalis*) represent frameshift indels (red triangles above the lines represent frameshift insertions, and red triangles represent frameshift deletions), and the green vertical line represents the M6 site in *O. sativa*.

The *PROG1* locus was analyzed by using the available genome sequences of eight AA genome *Oryza* species, i.e., *O. sativa* (Sasaki and International Rice Genome Sequencing Project, 2005), *O. glaberrima* (Wang et al., 2014), *O. longistaminata* (Zhang et al., 2015), *O. glumaepatula* (Zhang et al., 2014), *O. meridionalis* (Zhang et al., 2014), *O. rufipogon* (Stein et al., 2018), *O. nivara* (Stein et al., 2018), and *O. barthii* (Stein et al., 2018); one BB genome from *O. punctata* (Stein et al., 2018); and one FF genome from *O. brachyantha* (Chen et al., 2013). One non-*Oryza* Gramineae species, *Brachypodium distachyon* (Vogel et al., 2010), was used as an outgroup. Based on genomic alignment of the syntenic *PROG1* sequence, *O. rufipogon* shares homologous syntenic regions with the other species assessed in this study (Supplementary Table S1). Highly similar flanking genes, such as *Os07g0153300* and *Os07g0153400* in the 5′ upstream region of *PROG1* and *Os07g0154100* and *Os07g0154300* downstream were found in the eight AA genome *Oryza* species (Figure 1A). Although annotated genes in the neighboring loci were found, sequences homologous to *PROG1* but without traces of the short C2H2-type zinc-finger motif sequence (∼90 bp) were identified in the two distant species *O. brachyantha* and *B. distachyon*. The absence of the C2H2 motif in non-*Oryza-*species results in the lack of any homology to the peptide sequence. In the BB genome of *O. punctata*, the homologous *OPUNC07G03350.1* coding sequence, which was similar to that of *O. rufipogon* in length, showed four in-frame deletions, two in-frame insertions and more than 44 non-synonymous mutations (Supplementary Table S2). There was also a frameshift deletion in *O. barthii* or a frameshift insertion in both *O. nivara* and *O. meridionalis* (Figure 1B and Supplementary Figures S1, S2). Although the remaining three AA genomes (*O. sativa*, *O. longistaminata*, and *O. glumaepatula*) showed several mutations, they appeared to contain intact ORF sequences homologous to the *O. rufipogon PROG1* gene including a number of non-synonymous substitutions, two deletions and two insertions in *O. longistaminata* and one deletion and two insertions in *O. glumaepatula* (Supplementary Table S2 and Supplementary Figures S1, S2). Considering the fully prostrate architecture of *O. rufipogon* and the fact that the *O. rufipogon PROG1* gene is different from those in other *Oryza* species based on the sequence alignment, *PROG1* of *O. rufipogon* may have a highly effective function in controlling the prostrate phenotype. Therefore, we speculate that *PROG1* is a new gene, the ORF of which appeared in *O. punctata*, and may have undergone functionalization only in *O. rufipogon.*

### *PROG1* May Have Arose via Gene Duplication

**FIGURE 2:**
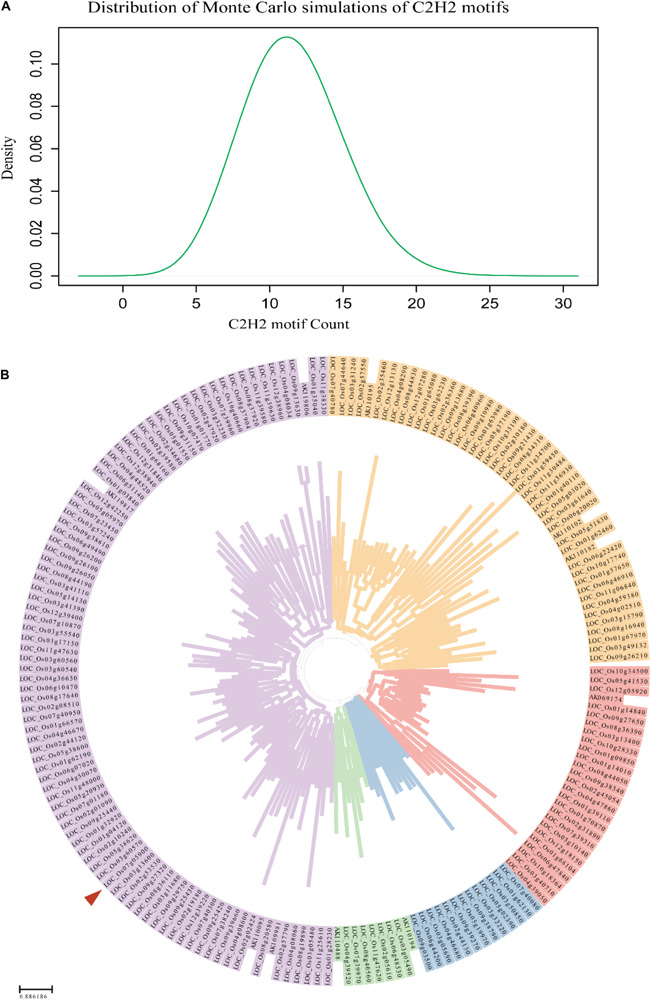
Monte Carlo simulation results for the random *de novo* origination of a C2H2 motif. **(A)** Distribution of Monte Carlo simulations of C2H2 motifs. **(B)** Clustering relationships of the 189 C2H2 transcription factor genes (Agarwal et al., 2007) in the genome of *O. sativa*. A red triangle indicates the *PROG1* (LOC_Os07g05900) of *O. sativa.*

Considering the sequence mutation data, *PROG1* appears to have arisen via gene duplication in *O. rufipogon*. To test this hypothesis, the possibility that C2H2 genes could originate in the rice genome by random chance was analyzed. In this analysis, Monte Carlo simulations of C2H2 motifs with repeated sampling for 100,000 iterations were conducted, and the expected number of C2H2 motifs was compared with the observed number (Figure 2A). Previous studies have identified 189 C2H2 transcription factors in the rice genome (Agarwal et al., 2007). Before clustering these genes, the conserved C2H2 motifs were deleted. Based on the computed distance matrices, the 189 genes were clustered into five groups (Figure 2B). This observation showed that these 189 C2H2 transcription factor genes were likely duplicated from five ancestral genes, or “starter gene.” We then performed Monte Carlo simulations with 100,000 iterations, using the number of starter genes as the expected number and the number of simulated C2H2 motifs as the observed number. We found that the chance of C2H2 motifs originating in the rice genome by random chance was 99.055%, suggesting that *ab initio* origination of a C2H2 gene is possible in the rice genome. These observations indicated that a C2H2 gene can be easily generated by random mutations in the rice genome. Moreover, the assessment of large gene families in the rice genome, such as *PROG1* (LOC_Os07g05900), showed an incidental gene duplication stemming from of a proto-*PROG1* gene (Figure 2B). The *RPAD* locus, which also participates in domesticated plant architecture in both Asian and African cultivated rice, harbors a tandem repeat of zinc-finger genes (including the *PROG1* gene) controlling plant architecture in wild rice (Wu et al., 2018). To determine whether the tandem repeats of zinc-finger genes originated from the same proto-*PROG1* gene, further phylogenetic tree analysis was performed, which clustered eight C2H2 genes, including *PROG1*, into one group (Supplementary Figure S3). This finding implies that the tandem repeats of the zinc-finger genes were produced by gene duplication. These results indicate that *PROG1* may have been produced from other C2H2-containing paralogous genes by gene duplication.

### *PROG1* of *O. rufipogon* Has a Strong Function in the Prostrate Plant Architecture

**FIGURE 3:**
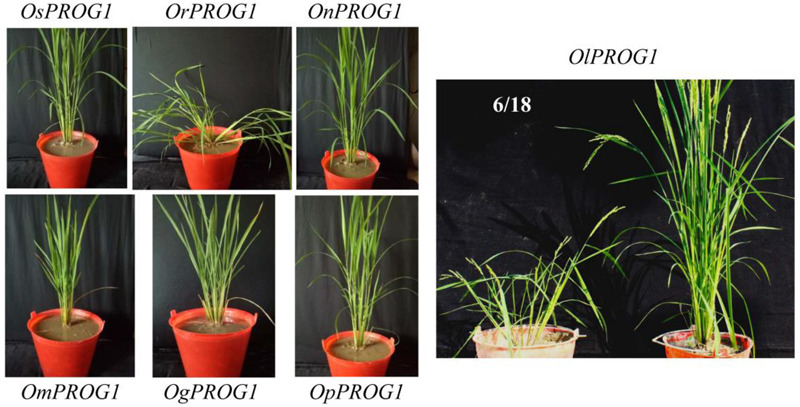
Role of *Oryza PROG1s* in controlling prostrate plant architecture driven by the *O. rufipogon PROG1* promoter. Transgenic verification of various *PROG1* alleles in *O. sativa* (Zhonghua 11). *OsPROG1*, *PROG1* from *O*. *sativa*. *OrPROG1*, *PROG1* from *O. rufipogon*. *OnPROG1*, *PROG1* from *O. nivara*. *OmPROG1*, *PROG1* from *O. meridionalis*. *OgPROG1*, *PROG1* from *O. glumaepatula*. *OpPROG1*, *PROG1* from *O. punctata*. *OlPROG1*, *PROG1* from *O. longistaminata*. Transgenic lines for each gene, *n* >15. The 6/18 ratio indicates that 6 lines exhibit a prostrate phenotype among 18 transgenic lines.

Interestingly, *PROG1* of *O. sativa* was found to be a pseudogene selected by a strong artificial selection (Jin et al., 2008; Tan et al., 2008; Xu et al., 2011; Huang et al., 2012). The transgenic experiments demonstrated that although *PROG1* in *O. sativa* has lost its function, it is actively involved in the prostrate phenotype of *O. rufipogon* (Figure 3); this finding is consistent with previous studies (Jin et al., 2008; Tan et al., 2008). All *PROG1*-homologous coding sequences from other *Oryza* species (except for that of *O. barthii*) driven by *PROG1* promoter of *O. rufipogon* were transformed into the Zhonghua 11 variety of *O. sativa* to verify their functions. The results suggested that *O. rufipogon PROG1* clearly has a function in producing a prostrate phenotype (Figure 3). The *PROG1* homologs of other *Oryza* species have no function or only weakly affect plant architecture. Interestingly, some transgenic lines expressing *O. longistaminata PROG1* showed divergent architecture (Figure 3).

**FIGURE 4:**
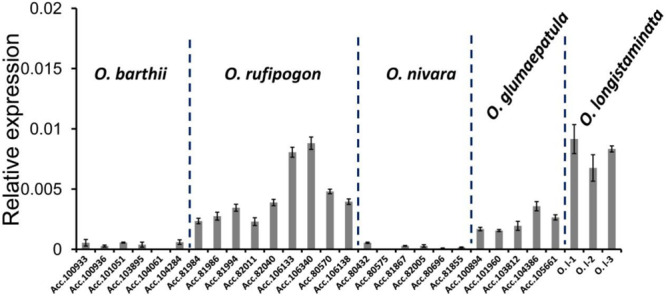
The different expression patterns of *PROG1* in different *Oryza* species. qRT-PCR analysis of *PROG1* in unelongated basal tiller internodes of various accessions (Supplementary Table S5) of *Oryza* species. Error bars indicate the standard deviation (SD) of three biological repeats.

Because *PROG1* functions in the tiller base of *O. rufipogon* and determines plant architecture, real-time PCR (qRT-PCR) was conducted to determine the expression of the *PROG1* homologs of *O. rufipogon*, *O. nivara*, *O. barthii*, *O. longistaminata*, and *O. glumaepatula*. This expression analysis showed that the *PROG1* gene was expressed in the unelongated basal tiller internodes of *O. rufipogon*, *O. longistaminata*, and *O. glumaepatula* (Figure 4). Previous studies also reported high expression of *PROG1* in the unelongated basal tiller internodes of *O. rufipogon* (Jin et al., 2008). However, an extremely low level of expression in the unelongated basal internodes was detected in *O. sativa* (Jin et al., 2008), *O. nivara*, and *O. barthii* (Figure 4). In two transcriptomes (leaf and panicle) of *O. nivara* and *O. barthii* (Wang et al., 2014), only an extremely low level of expression of a pseudo-*PROG1* gene was detected in the panicle transcriptome of *O. barthii*, and no expression was detected in *O. nivara*. In eight transcriptomes from the rhizome, stem, rhizome tips, stem tips, stamens, pistils, hybrid line stamens, and hybrid line pistils of *O. longistaminata* (Zhang et al., 2015), low levels of expression were detected in only the rhizome tips and stamens (Supplementary Table S3). The transgenic test in this study revealed *PROG1* as an expressible pseudogene in *O. glumaepatula* that possibly plays a role in regulating rhizome transverse elongation in *O. longistaminata*. In the NCBI database of the BB genome of *O. punctata*, no expression of *OPUNC07G03350.1* was detected in the two transcriptomes (root and panicle) (Supplementary Table S3). In the two transcriptomes of *O. brachyantha* (Chen et al., 2013) and 11 transcriptomes of *B. distachyon* (two transcriptomes from 20-day leaves, two transcriptomes from embryos 25 days after pollination, endosperm 25 days after pollination, early inflorescence, emerging inflorescences, pistils, seeds 5 days after pollination, anthers, and seeds 10 days after pollination) (Davidson et al., 2012), no expression of annotated non-homologous genes at the *PROG1* locus was detected (Supplementary Table S4). Except in *O. rufipogon*, the collective expression results of the *Oryza* species did not indicate the *PROG1* locus as a unique gene related to tiller development. The very low expression of the *PROG1* locus in the various tissues of different *Oryza* species suggests the possibility that the *PROG1* gene evolved as a functional gene until the appearance of the most recent *O. rufipogon* ancestor.

### The Young *PROG1* Gene Underwent Strong Natural Selection in *O. rufipogon*

**FIGURE 5:**
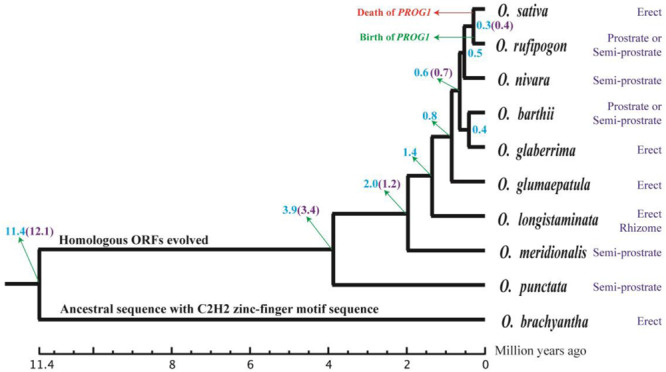
Evolution of plant architecture in *Oryza* species. The evolutionary process of the young orphan gene *PROG1* was labeled on the corresponding branches, and the plant architecture phenotypes of 10 *Oryza* species are shown to the right of the phylogenetic tree. The estimated divergence time is labeled at each node. The numbers marked with blue were estimated with whole-genome data, and those marked with purple are from TimeTree.

Using the whole genome, a phylogenetic tree of 10 *Oryza* species was constructed, and their divergence times were estimated using all the single-copy genes in their genomes. The phylogenetic results of this study are similar to those shown in TimeTree (Zhu and Ge, 2005; Hedges et al., 2006; Figure 5). For instance, 11.4 Mya in TimeTree and 12.1 Mya in our results were the suggested divergence times of *O. brachyantha*. Likewise, *O. rufipogon* diverged from other species at 0.4 Mya in TimeTree and 0.3 Mya in this study. The first homologous ORF of *PROG1* appeared in the BB genome of *O. punctata* at ~3.4–3.9 Mya. These results suggest that the *PROG1* gene encoding a plant architecture regulatory protein in *O. rufipogon* was notably young (0.3–0.4 Mya).

To examine *PROG1* as a gene controlling the prostrate plant architecture in *O. rufipogon*, we grew *O. rufipogon*, *O. nivara*, *O. glaberrima*, *O. barthii*, *O. longistaminata*, *O. glumaepatula*, and *O. punctata* to check their architectures. During this architecture assessment, a fully prostrate plant architecture was observed only in *O. rufipogon*. The other *Oryza* species showed non-prostrate or semi-prostrate architectures that were either strictly erect or semi-erect with some angled tillers (Supplementary Table S5 and Figure 5). The published population data of *PROG1* in *O. rufipogon* (Tan et al., 2008) were analyzed to assess the hypothesis of a selective sweep of *PROG1* in *O. rufipogon*. Tajima’s *D* test detected a significant departure from neutrality in this gene (3.03762, *P* < 0.001) and suggested strong natural selection on *PROG1* in *O. rufipogon*. Furthermore, Fu and Li’s test was conducted using the outgroup sequences of *PROG1* loci from four *Oryza* species (*O. punctata*, *O. glumaepatula*, *O. longistaminata*, and *O. sativa*). A significant signal of positive selection was detected in *O. rufipogon* in relation to either of the outgroup sequences, with a *p* < 0.05 for both the D and F statistics of Fu and Li’s test (Supplementary Table S6). These results provide evidence that the prostrate architecture is a derived trait that has undergone strong natural selection in *O. rufipogon* and that the trait is conferred or enhanced by the *PROG1* gene.

## Discussion

Plant architecture plays important roles in plant survival and adaptation to diverse conditions. It has been reported that *O. rufipogon* inhabits swamps with moderately deep water (Grillo et al., 2009), and thus, the prostrate plant architecture that evolved in *O. rufipogon* likely allowed it to spread across the water to achieve greater access to light and chemical nutrients and confers structural tenacity on variable surfaces. Our study demonstrated that the critical *PROG1* gene locus, which regulates plant architecture in *O. rufipogon*, emerged through a process of *de novo* origination from 3.4 to 3.9 Mya in *O. punctata* and evolved into a functional gene strongly affecting phenotype in *O. rufipogon* between 0.3 and 0.4 Mya. Because *PROG1* evolved recently, the adaptive evolution and selection of *PROG1* in *O. rufipogon* populations may still be ongoing. Published population data for *PROG1* in *O. rufipogon* (Tan et al., 2008) were analyzed to examine the hypothesis of a selective sweep of *PROG1* in *O. rufipogon*. Tajima’s *D* test revealed a significant departure from neutrality for this gene (3.03762, *P* < 0.001) and suggested the existence of strong natural selection on *PROG1* in *O. rufipogon*. The sequence evolution in the *PROG1* gene, together with the derived prostrate plant architecture of *O. rufipogon*, suggests strong adaptive evolution that started with the fixation of this young gene in ancestral *O. rufipogon* populations and continues in an extant population.

Causative mutations including SNPs (Konishi et al., 2006; Li et al., 2006; Lin et al., 2007), structural variations such as small indels (Sweeney et al., 2006; Hua et al., 2015; Bessho-Uehara et al., 2016; Jin et al., 2016), large structural variations (Wu et al., 2018), and mobile DNA elements (Studer et al., 2011) play important roles during crop domestication and usually result in dysfunctions and/or alterations of the expression patterns of domestication-related genes. New genes can be produced in multiple ways, including gene duplication and *de novo* origination from previously non-coding sequences (Ding et al., 2012). The *PROG1* gene may have originated in the BB genome of *O. punctata* as a C2H2 gene with an unknown function and been neo-functionalized by gaining a function related to prostrate architecture in *O. rufipogon*. Similar tandem repeats of zinc-finger protein-coding genes have been found in the collinear chromosomal region of the *RPAD* locus and might be recognized as an ancient zinc-finger gene cluster with a conserved functional role in the regulation of plant growth habits. Because several C2H2 genes, including *PROG1*, *ZnF5*, *ZnF7*, and *ZnF8*, function in the control of the prostrate-growth trait (Wu et al., 2018), causative mutations cannot be found in *PROG1*. A second hypothesis is that this gene was not a functional gene before the evolution of *O. rufipogon*, which is supported by evidence from transgenic experiments, gene expression, phenotyping and assessments of repeated ORF disruptions and remarkable sequence variability in different species.

Interestingly, the *PROG1* expression level in *O. longistaminata* is as high as that in *O. rufipogon*. Considering that some transgenic lines expressing *O. longistaminata PROG1* exhibit a semi-prostrate phenotype, whether *O. longistaminata PROG1* functions in the lateral elongation of rhizomes needs further investigation. The deletion site at the *RPAD* locus was also a target of artificial selection during domestication in both Asian and African rice (Wu et al., 2018). Although it remains unclear whether the causative mutations within the *PROG1* promoter and coding sequence are associated with prostrate function, variations in protein sequence and expression were selected by rice breeding (Jin et al., 2008; Tan et al., 2008).

Domestication-related genes such as *fw2.2*, *fascinated* (*fas*), *teosinte glume architecture* (*tga1*), *teosinte branched1* (*tb1*), *IPA1*, and *OsTb2*, are responsible for agricultural advances and morphological improvements during rice, tomato and maize domestication (Doebley et al., 1997; Wang et al., 1999, 2005; Frary et al., 2000; Cong et al., 2008; Lu et al., 2013; Lyu et al., 2020). Mutations in genes such as *tb1*, *tin1*, *IPA1*, and *OsTb2* brought about the change from wild Mexican grass (*teosinte*) and *O. rufipogon* to the cultivate type architecture mainly for branch numbers in maize and rice domestication (Doebley et al., 1997; Wang et al., 1999; Lu et al., 2013; Zhang X. et al., 2019; Lyu et al., 2020). Tiller angle controlling genes *TAC1* and *LA1* (*LAZY1*) and *PROG1* also play important roles in rice architecture for high-density planting during rice domestication (Li et al., 2007; Yoshihara and Iino, 2007; Yu et al., 2007; Jin et al., 2008; Tan et al., 2008; Jiang et al., 2012; Lyu et al., 2020). These observations lead us to speculate that these genes contributed to survival and adaptation in the wild ancestor species. A similar transition from prostrate to erect growth occurred during the domestication of wheat (Waisel, 1987). This finding provides another piece of evidence that a gene influencing plant architecture can be adaptive in the ancestor species, whereas its mutation can improve plant architecture and yield in domesticated crops (Doebley et al., 1997; Clark et al., 2006; Wang et al., 2018).

## Conclusion

*PROG1* is a new functional gene that was likely generated through gene duplication, and its predicted young age could be a result of a loss of sequence identity due to a high level of substitution in an ancient gene. Natural selection in a swamp habitat led to *PROG1* functionalization to produce fully prostrate plant architecture, and artificial domestication aimed at maximizing yield via the high-density planting of rice with an erect plant architecture led to the pseudogenization of this gene and deletion of the *RPAD* locus.

## Data Availability Statement

Publicly available datasets were analyzed in this study. This data can be found here: SRR1171006 and SRR1171007.

## Author Contributions

LH and YZ designed and performed the experiments, designed the experiments, and wrote the manuscript. HL and JW analyzed the sequence data. RZ and YL generated the transgenic materials. YB and MN provided assistance in vector construction. SZ, GH, BC, QH, YG, and JZ provided assistance in the phenotyping experiment. GM revised the manuscript. All authors read and approved the final manuscript.

## Conflict of Interest

YL and YZ were employed by BGI-Baoshan. The remaining authors declare that the research was conducted in the absence of any commercial or financial relationships that could be construed as a potential conflict of interest.
