# Supplementary material for: Evolution of Plant Architecture in Oryza Driven by the PROG1 Locus
Source: Front Plant Sci. 2020 Jun 23;11:876. doi: 10.3389/fpls.2020.00876 (PMC7325765; doi:10.3389/fpls.2020.00876)
Supplement: Supplementary file 1 [file Data_Sheet_1.docx]

**Table S1. The annotated gene IDs at the *PROG1* locus in different species.**

| **Species** | **Gene ID** | **Genome version** |
| --- | --- | --- |
| *O. sativa* | Os07g0153600 | IRGSP-1.0 |
| *O. rufipogon* | ORUFI07G03100.1 | PRJEB4137 |
| *O. nivara* | ONIVA07G01430.1 | AWHD00000000 |
| *O. longistaminata* | Olong01m10014772.1 | *Oryza_longistaminata*_v1.0 |
| *O. barthii* | OBART07G03450.1 | [ABRL00000000](http://www.ebi.ac.uk/ena/data/view/GCA_000182155.2) |
| *O. glaberrima* | - | [AGI1.1](http://www.ebi.ac.uk/ena/data/view/GCA_000147395.1) |
| *O. meridionalis* | OMERI07G02110.1 | *Oryza_meridionalis*_v1.3 |
| *O. glumaepatula* | OGLUM07G02740.1 | ALNU02000000 |
| *O. punctata* | OPUNC07G03350.1 | AVCL00000000 |
| *O. brachyantha* | OB07G12600.1 | [*Oryza_brachyantha*.v1.4b](http://www.ebi.ac.uk/ena/data/view/GCA_000231095.2) |
| *B. distachyon* | Bradi1g58540.1 | [v1.0](http://www.ebi.ac.uk/ena/data/view/GCA_000005505.1) |

**Table S2. Mutations in *O. sativa*, *O. longistaminat*a, *O. glumaepatula* and *O. punctata* compared to *O. rufipogon PROG1.***

| **Species** | **Number of non-synonymous substitution** | **Number of inframe deletion** | **Number of inframe insertion** |
| --- | --- | --- | --- |
| *O. sativa* | 1 | 0 | 0 |
| *O. longistaminata* | 13 | 2 | 2 |
| *O. glumaepatula* | 13 | 1 | 2 |
| *O. punctata* | 44 | 4 | 2 |

**Table S3. Expression level of *PROG1*-homologous sequences in 22 transcriptomes of *O. sativa*, *O. longistaminata*, *O. nivara*, *O. barthii* and *O. punctata.***

| **Species** | **Tissues** | ***ACTIN1*** | ***PROG1*** |
| --- | --- | --- | --- |
| *O. sativa* | Callus | 203.166 | 0 |
|  | Booting panicles | 289.039 | 0 |
|  | Seeding shoot | 31.1715 | 0 |
|  | Tillering leaf | 2.62667 | 0 |
|  | Flowering panicle | 15.9779 | 0 |
|  | Filling leaf | 5.27607 | 0 |
|  | Filling panicle | 33.4219 | 0 |
| *O. longistaminata* | Rhizome | 69.2818 | 0 |
|  | Rhizome-tips | 274.981 | 5.192 |
|  | Stem | 205.696 | 0 |
|  | Stem-tips | 165.639 | 0 |
|  | Stamens | 707.297 | 4.621 |
|  | Pistils | 126.281 | 0 |
|  | Hybrid stamens | 670.686 | 0 |
|  | Hybrid pistils | 175.543 | 0 |
| *O. nivara* | Leaf | 22.582 | 0 |
|  | Panicle | 354.586 | 0 |
| *O. bathii* | Leaf | 33.823 | 0 |
|  | Panicle | 263.633 | 0.091 |
| *O. punctata* | panicle | 296.896 | 0 |
|  | root | 15.5774 | 0 |

Numbers represent the FPKM value.

**Table S4. Expression levels of *PROG1*-homologous sequences in 14 transcriptomes of the outgroup species *B. distachyon* and *O. brachyantha*.**

| **Species** | **Tissues** | ***ACTIN1*** | ***PROG1*** |
| --- | --- | --- | --- |
| *B. distachyon* | Bd21 20 Day Leaves | 17.6588 | 0 |
|  | Bd21 Early Inflorescence | 546.674 | 0 |
|  | Bd21 Emerging Inflorescence | 492.518 | 0 |
|  | Bd21 Pistil | 315.595 | 0 |
|  | Bd21 Embryo 25 Days After Pollination | 132.553 | 0 |
|  | Bd21 Seed 5 Days After Pollination | 531.068 | 0 |
|  | Bd21 Anther | 3978.08 | 0.354033 |
|  | Bd21 Seed 10 Days After Pollination | 302.519 | 0 |
|  | Bd21 Endosperm 25 Days After Pollination | 68.1136 | 0 |
|  | Bd21 20 Day Leaves | 17.919 | 0 |
|  | Bd21 Embryo 25 Days After Pollination | 275.037 | 0.717164 |
| *O. brachyantha* | *Oryza brachyantha* IRGC101232 | 210.22 | 0 |
|  | *Oryza brachyantha* IRGC101232 | 54.595 | 0 |

Numbers represent the FPKM value.

**Table S5. Information list of *Oryza* species used in this study.**

| ***Oryza* species** | **Accession NO.** | **Source** | **Plant architecture** |
| --- | --- | --- | --- |
| *O.rufipogon* | Acc.80433 | India | prostrate |
| *O.rufipogon* | Acc.80742 | Myanmar | prostrate |
| *O.rufipogon* | Acc.81801 | Indonesia | prostrate |
| *O.rufipogon* | Acc.81976 | Indonesia | prostrate |
| *O.rufipogon* | Acc.81984 | Laos | Semi-prostrate |
| *O.rufipogon* | Acc.81986 | Cambodia | Semi-prostrate |
| *O.rufipogon* | Acc.81994 | Papua New Guinea | prostrate |
| *O.rufipogon* | Acc.82011 | India | prostrate |
| *O.rufipogon* | Acc.82040 | Thailand | prostrate |
| *O.rufipogon* | Acc.100639 | Taiwan | Semi-prostrate |
| *O.rufipogon* | Acc.103308 | Taiwan | Semi-prostrate |
| *O.rufipogon* | Acc.105832 | Thailand | Semi-erect |
| *O.rufipogon* | Acc.106133 | India | Semi-prostrate |
| *O.rufipogon* | Acc.106138 | India | Semi-prostrate |
| *O.rufipogon* | Acc.106340 | Myanmar | prostrate |
| *O.rufipogon* | Acc.80570 | India | prostrate |
| *O.rufipogon* | Acc.102159 | Thailand | Semi-prostrate |
| *O.rufipogon* | AYTY9602 | Thailand | Semi-prostrate |
| *O.rufipogon* | PCR9607 | Thailand | Semi-erect |
| *O.rufipogon* | Acc.105832 | Bgo2,1998 gkanlaus | prostrate |
| *O.rufipogon* | Acc.106138 | Bgo2,1999 gkanlaus | Semi-prostrate |
| *O.rufipogon* | Acc.106138 | Bgo2,2001 gkanlaus | prostrate |
| *O.nivara* | Acc.80581 | India | Semi-prostrate |
| *O.nivara* | Acc.80611 | India | prostrate |
| *O.nivara* | Acc.80625 | India | prostrate |
| *O.nivara* | Acc.80681 | India | Semi-prostrate |
| *O.nivara* | Acc.81867 | India | Semi-prostrate |
| *O.nivara* | Acc.80696 | India | Semi-prostrate |
| *O.barthii* | Acc.100921 |  | Semi-prostrate |
| *O.barthii* | Acc.100927 | Sierra Lenone | prostrate |
| *O.barthii* | Acc.100933 | Sudan | Semi-prostrate |
| *O.barthii* | Acc.100936 | Niger | Semi-prostrate |
| *O.barthii* | Acc.101051 | Africa | Semi-prostrate |
| *O.barthii* | Acc.101196 | Cameroom | prostrate |
| *O.barthii* | Acc.103895 | Senegal | Semi-prostrate |
| *O.barthii* | Acc.104061 | Niger | Semi-prostrate |
| *O.barthii* | Acc.104102 | Chad | Semi-prostrate |
| *O.barthii* | Acc.104132 | Cameroom | Semi-prostrate |
| *O.barthii* | Acc.104284 | Mali | Semi-prostrate |
| *O.barthii* | Acc.104103 | Chad | Semi-prostrate |
| *O.barthii* | Acc.105507 | Mali | prostrate |
| *O.glumaepatula* | Acc.100894 | Cuba | Semi-prostrate |
| *O.glumaepatula* | Acc.103812 | Brazil | Semi-prostrate |
| *O.glumaepatula* | Acc.105661 | Brazil | Semi-prostrate |
| *O.meridionalis* | Acc.103322 | Australia | Semi-prostrate |
| *O.meridionalis* | Acc.104498 | Australia | Semi-prostrate |
| *O.meridionalis* | Acc.105298 | Australia | Semi-prostrate |
| *O.punctata* | Acc.100892 |  | Semi-prostrate |

**—**—indicate that samples which have no Accession NO.

**Table S6. Fu and Li’s test on the *PROG1* gene using *O. rufipogon* population data [17] with 4 species as outgroups.**

| **Outgroup** | **D test statistics** | ***P* value** | **F test statistics** | ***P* value** |
| --- | --- | --- | --- | --- |
| *O. sativa* | 1.7408 | *P* < 0.02 | 2.61434 | *P* < 0.02 |
| *O. longistaminata* | 1.71654 | *P* < 0.02 | 2.5738 | *P* < 0.02 |
| *O. glumaepatula* | 1.76324 | *P* < 0.02 | 2.65162 | *P* < 0.02 |
| *O. punctata* | 1.66162 | *P* < 0.05 | 2.48101 | *P* < 0.02 |

**
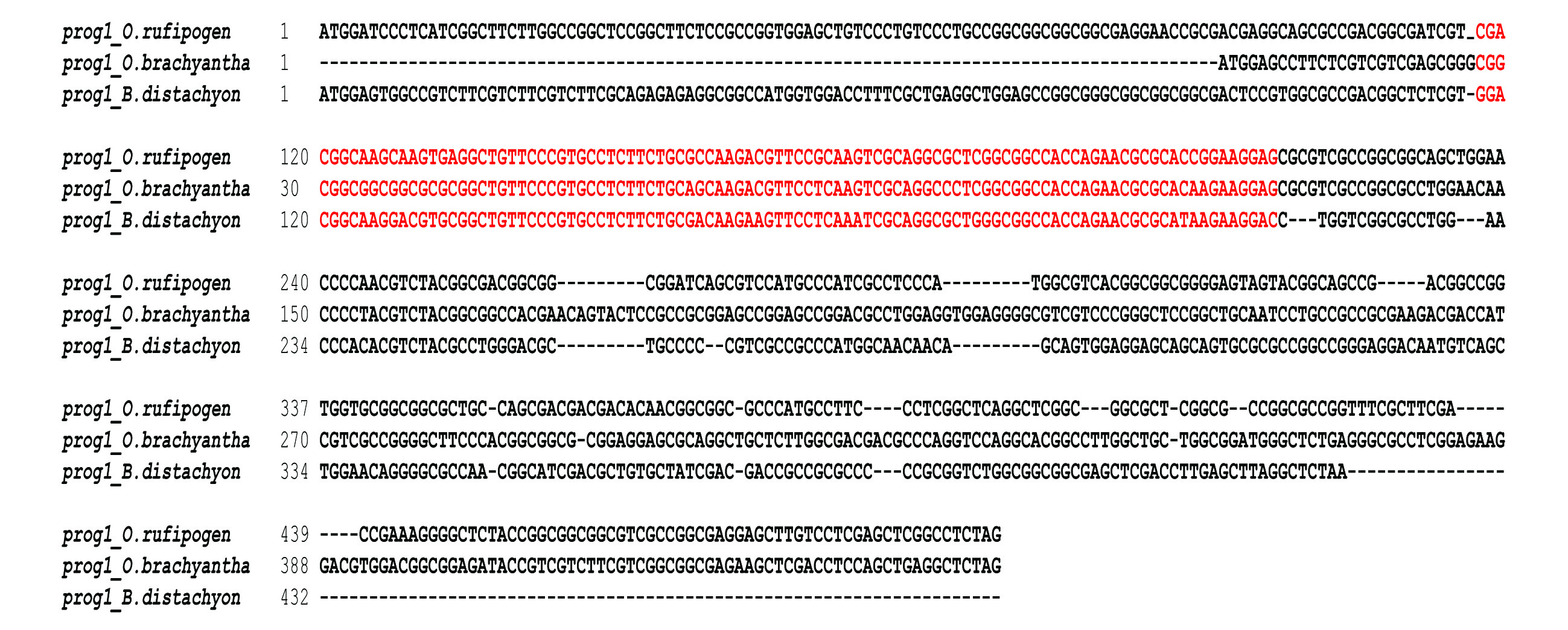
**

**Figure S1. Alignment of the DNA sequence of the *PROG1* locus in *O. rufipogon*, *O. brachyantha* and *B. distachyon*. Except for the C2H2 motif, which is marked with red, no obvious homology was identified.**

**
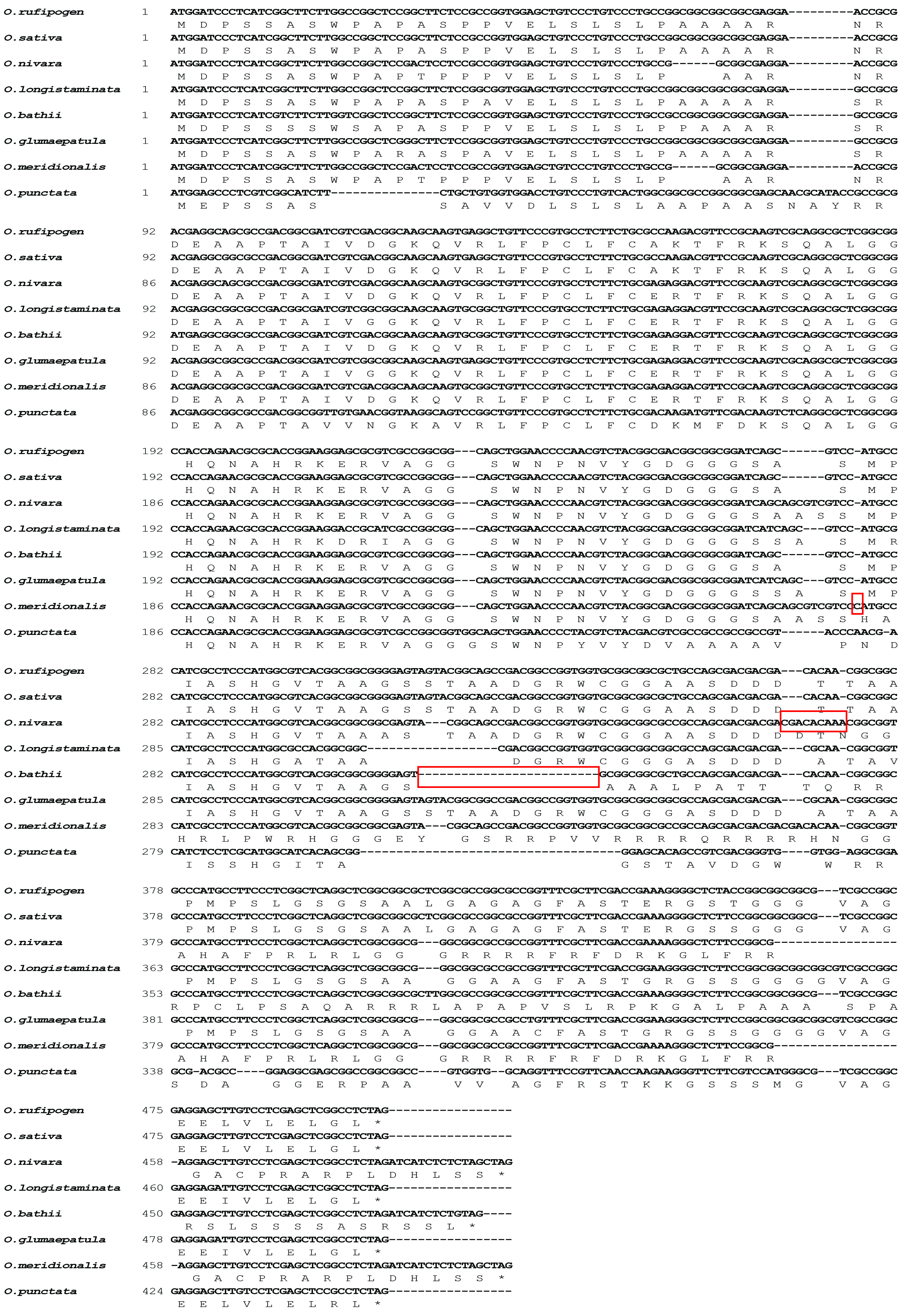
**

**Figure S2. Alignment of DNA sequences and putative peptide sequences of the *PROG1* locus in 8 *Oryza* species. The red blocks represent the frameshift insertions in *O. meridionalis* and *O. nivara* and the deletion in *O. barthii*.**


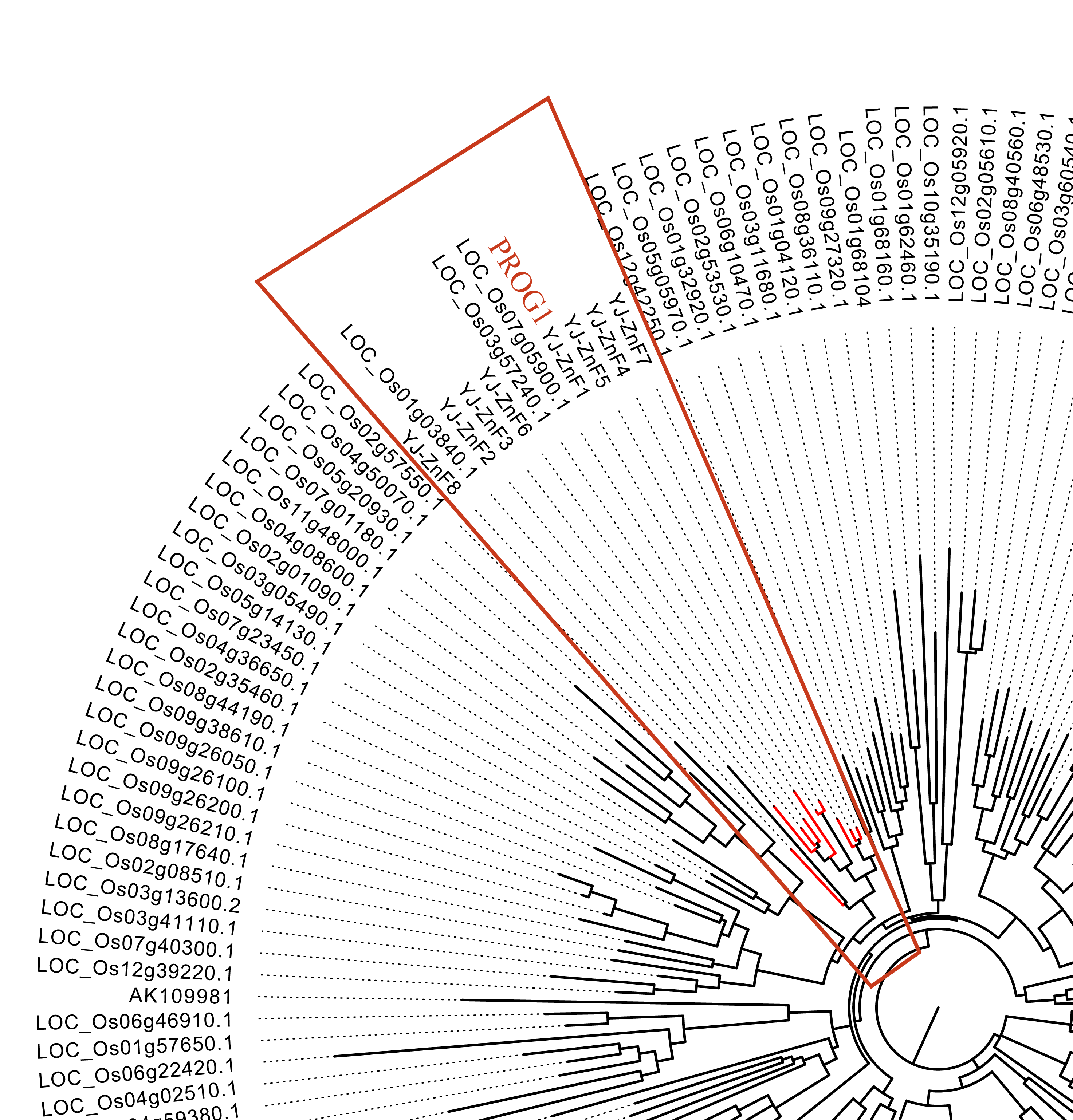
 **Figure S3. 8 C2H2 genes including PROG1 in the RICE PLANT ARCHITECTURE DOMESTICATION (RPAD) locus stemmed from one proto-PROG1 gene.**
